# Knotify_V2.0: Deciphering RNA Secondary Structures with H-Type Pseudoknots and Hairpin Loops

**DOI:** 10.3390/genes15060670

**Published:** 2024-05-23

**Authors:** Angelos Kolaitis, Evangelos Makris, Alexandros Anastasios Karagiannis, Panayiotis Tsanakas, Christos Pavlatos

**Affiliations:** 1 School of Electrical and Computer Engineering, National Technical University of Athens, 9 Iroon Polytechniou St., 15780 Athens, Greece; akolaitis@mail.ntua.gr (A.K.); vmakris@mail.ntua.gr (E.M.); alankaragiannis@mail.ntua.gr (A.A.K.); panag@cs.ntua.gr (P.T.); 2 Hellenic Air Force Academy, Dekelia Air Base, Acharnes, 13671 Athens, Greece

**Keywords:** RNA, pseudoknot motif, bulges, hairpins, syntactic pattern recognition

## Abstract

Accurately predicting the pairing order of bases in RNA molecules is essential for anticipating RNA secondary structures. Consequently, this task holds significant importance in unveiling previously unknown biological processes. The urgent need to comprehend RNA structures has been accentuated by the unprecedented impact of the widespread COVID-19 pandemic. This paper presents a framework, Knotify_V2.0, which makes use of syntactic pattern recognition techniques in order to predict RNA structures, with a specific emphasis on tackling the demanding task of predicting H-type pseudoknots that encompass bulges and hairpins. By leveraging the expressive capabilities of a Context-Free Grammar (CFG), the suggested framework integrates the inherent benefits of CFG and makes use of minimum free energy and maximum base pairing criteria. This integration enables the effective management of this inherently ambiguous task. The main contribution of Knotify_V2.0 compared to earlier versions lies in its capacity to identify additional motifs like bulges and hairpins within the internal loops of the pseudoknot. Notably, the proposed methodology, Knotify_V2.0, demonstrates superior accuracy in predicting core stems compared to state-of-the-art frameworks. Knotify_V2.0 exhibited exceptional performance by accurately identifying both core base pairing that form the ground truth pseudoknot in 70% of the examined sequences. Furthermore, Knotify_V2.0 narrowed the performance gap with Knotty, which had demonstrated better performance than Knotify and even surpassed it in Recall and F1-score metrics. Knotify_V2.0 achieved a higher count of true positives (*tp*) and a significantly lower count of false negatives (*fn*) compared to Knotify, highlighting improvements in Prediction and Recall metrics, respectively. Consequently, Knotify_V2.0 achieved a higher F1-score than any other platform. The source code and comprehensive implementation details of Knotify_V2.0 are publicly available on GitHub.

## 1. Introduction

RNA, which is a pivotal molecule in various biological processes, plays a crucial role in decoding genetic information. The transcription of DNA molecules into mRNA facilitates the translation process, ultimately leading to protein production—an essential aspect known as the “central dogma” of molecular biology [1]. Beyond this fundamental function, RNA is intricately involved in various biological phenomena, including site recognition, catalysis, and the regulation of gene expression [2,3]. Except for mRNA, noncoding RNAs fulfill functions beyond protein encoding, necessitating a detailed analysis of these molecules. To comprehend RNA’s functions, predicting its 3D structure becomes imperative [4,5]. While techniques like X-ray crystallography [6] and nuclear magnetic resonance [7] offer ways to determine tertiary structures, focus on the prediction of a simplified 2D representation, termed the secondary structure, has intensified. The secondary structure comprises A-U (Adenine-Uracil), C-G (Cytosine-Guanine), and G-U (Cytosine-Uracil) pairs forming duplex regions, along with unpaired bases that create motifs such as loops, bulges, and hairpins. The accurate identification of these structural elements serves as a crucial foundation for understanding RNA operations.

Recent methods for predicting the secondary structure of RNA rely primarily on scoring functions that can utilize algorithms based on artificial intelligence (AI), probability, or thermodynamics. Justyna et al. [8] have conducted an extensive benchmarking of AI methods in this domain, shedding light on the efficacy of these approaches. Many of these techniques use dynamic programming that is improved using experimentally determined parameters, and they all follow Zuker’s variation of minimal free energy algorithm [9]. Using dynamic programming, the widely-used Nussinov method has correctly predicted a significant number of base pairs [10], demonstrating improved performance when integrated into more sophisticated algorithms [11]. To address the prediction issue, modern approaches use machine learning, statistical techniques, heuristic algorithms, syntactic pattern recognition, integer programming, and stochastic methods. A thorough examination of the relevant literature is available in Section 2. Within the realm of RNA secondary structure, anticipating pseudoknots represents the most formidable challenge. Although various techniques can reliably predict common motifs such as stems, bulges, internal loops, hairpins, and multibranch loops in the RNA secondary structure, pseudoknot prediction proves to be a complex task. This challenge extends beyond prediction to encompass proper annotation within the 3D structure of RNAs, as demonstrated by Smit et al. [12] and Antczak et al. [13]. Dynamic programming and minimum free energy algorithms are not inherently equipped to manage the interconnections involved in pseudoknots. In addition, computational demands escalate exponentially with the length of the RNA, adding to the complexity of the problem. To address the need for accurate pseudoknot prediction, our research has led to the development of a platform named Knotify_V2.0. Knotify_V2.0 can predict H-type pseudoknots, with additional motifs such as internal loops, bulges, and hairpins in their loops. This platform maintains execution time efficiency while achieving accuracy on par with popular techniques.

In this study, a focus on the more complex motifs of an H-type pseudoknot is presented. According to the definition in [14], the H-type pseudoknot has two loops of any length and two stems. In those loops, a variety of simple or complex motifs are formed and affect the stability and functionality of the molecule. A usual and important example of that is hairpin loops. Hairpin loops or hairpins are motifs in RNA, characterized by stem-loop structures, which are indispensable components influencing diverse biological processes, serving as key regulators of RNA stability, function, and interactions. With a double-stranded stem formed by complementary base pairing and a terminal loop, these motifs are central to gene regulation, RNA splicing, and understanding disease mechanisms. In the realm of gene expression, hairpin structures in mRNA profoundly impact translational efficiency, particularly near the 5′ untranslated region (UTR). Studies, such as [15], emphasize that stable hairpins can hinder ribosome binding, regulating protein synthesis and emphasizing the crucial role of RNA secondary structures in controlling gene expression at the translational level.

Furthermore, as highlighted in [16], hairpin motifs play a critical role in RNA splicing, a fundamental process in the maturation of mRNA. The spliceosome, a complex molecular machine, relies on specific hairpin structures in pre-mRNA. These interactions are pivotal for accurate splicing, and alterations in hairpin structures can lead to splicing errors and various genetic disorders. Additionally, in the context of RNA interference (RNAi), hairpin RNA structures serve as precursors to microRNAs (miRNAs) and small interfering RNAs (siRNAs), guiding the RNA-induced silencing complex (RISC) to target mRNAs [17]. The involvement of hairpin structures in the pathogenesis of diseases, shown in [18], further underscores their importance. For instance, expanded hairpin structures formed by repeat expansions in RNA can contribute to neurodegenerative disorders.

Recognizing the multifaceted roles of hairpin motifs, it becomes imperative to incorporate them into pseudoknot prediction algorithms. The versatility of hairpin motifs, pivotal in gene regulation, splicing, and disease mechanisms, makes their inclusion crucial for a comprehensive understanding of the secondary structures of RNA. By incorporating the influence of hairpin structures, pseudoknot prediction algorithms can achieve greater accuracy and relevance, especially in scenarios where these motifs play a significant role in shaping the intricate landscape of RNA secondary structures.

In the present study, we propose an updated version of Knotify+ [19], the latest version of Knotify [20] system, that extends its predictive capabilities to include except the detection of bulges and internal loops [21], a novel feature for predicting hairpins within the loops of the H-type pseudoknots. Initiating the process, a parser is employed to analyze the RNA sequence, generating a comprehensive list of potential core stems for a pseudoknot. Subsequently, these core stems are augmented with potential base pairs located in proximity to the two essential stems of the pseudoknot. Interestingly, the method is made to detect possible hairpins surrounding these structures in addition to bulges and interior loops. A collection of candidates is determined by counting the maximum number of base pairs to find the optimal tree; the structure with the least amount of free energy is then chosen. With the latest update, Knotify_V2.0 can now predict and identify even more intricate motifs, such as hairpins, while retaining the same degree of complexity. Although there are more computational steps involved, the effect on the algorithm’s runtime is considered reasonable, compared with the increased accuracy in the prediction task.

## 2. Related Work

Many algorithms integrate dynamic programming into their processes to predict RNA’s most probable secondary structure while aiming to reduce free energy [22,23]. Some methods have also concentrated on pseudoknot prediction, utilizing entropy, stability, and the principle of minimum free energy [24]. The classification of this issue as NP-complete [25] has spurred the creation of stochastic and heuristic strategies [26,27,28]. For example, Knotty [29] uses the Chen–Condon–Jabbari (CCJ) algorithm [30] with sparsification for pseudoknot prediction. ProbKnot [31], on the other hand, estimates the base pair probabilities for regions without pseudoknots to infer the secondary structure with the highest expected accuracy. IPknot [32] outperforms earlier models by combining integer programming with base pair probability assessment. Its updated version [33] employs the LinearPartition model and pseudo-expected accuracy to predict secondary structures including pseudoknots efficiently, even for long sequences, though it still faces challenges in improving accuracy.

Alternative methods like Pfold [34,35], PPfold [36], and RNA-Decoder [37] employ Stochastic Context-Free Grammar (SCFG) to predict RNA’s secondary structure. These techniques excel in identifying patterns within structures, allowing for refinement through the strategic weighting of rules. Similarly, SCFG-based models such as Contrafold [38], Evfold [39], Infernal [40], and Oxfold [41] have been developed, emphasizing the importance of effectively integrating grammar with computational approaches. This includes the use of heuristic and probabilistic algorithms, strategies for minimizing free energy, maximizing base pairings, and calculating base pairing probabilities, in conjunction with other computational and biological considerations. Achieving an accurate prediction of RNA secondary structure necessitates a harmonious balance between these elements. Consistent with these findings, we introduce a grammar-based framework that optimizes base pairing and energy efficiency to improve prediction accuracy similarly to [42]. This framework fundamentally relies on Context-Free Grammar (CFG) as its operational model. In addition, it’s worth mentioning that some prediction methods allow adding experimental data to improve and/or guide the RNA secondary structure prediction, the following works address this issue [43,44].

Machine learning techniques have been introduced as a solution to discover hidden patterns within data, using supervised and unsupervised learning approaches in training data sets. These methods often rely on large datasets, especially when employing deep learning technologies, to ensure the model is well-trained and to prevent overfitting. For instance, a study introduced in [45] utilized deep learning alongside tertiary constraints for this purpose, while another research [11] used bidirectional Long Short-Term Memory (LSTM) networks combined with an improved base-pair maximization principle (IBPMP) for accurate base pair selection and optimal structure prediction. The 2dRNA [46] project introduced a two-stage deep neural network that feeds into a U-net architecture, where a bidirectional LSTM first encodes the data into a higher dimension before a fully connected network decodes them into a dot-bracket structure. Additionally, ATTfold [47] employs deep learning with an attention mechanism as an encoder to predict secondary structures, including pseudoknots. This model encodes a base pairing score matrix, which a Convolutional Neural Network (CNN) then decodes, aligning with strict biological principles to eliminate rare structures of the molecule in nature, adhering to established folding rules.

## 3. The Background Theory

In this section, we cover the foundations of several key theoretical ideas, such as RNA, hairpins, pseudoknots, and parsers. These data are essential to comprehend the methods suggested in Section 4.

### 3.1. RNA

All living cells contain RNA, a nucleic acid with structural similarities to DNA. In contrast to DNA, RNA is typically single-stranded and folds into the A-U and G-C Watson-Crick-Franklin base pairs, as well as the less common G-U wobble-base pair [48]. The secondary structure of this structure is essential to understanding many biological processes. RNA, including mRNA, tRNA, and rRNA, involved in protein synthesis, is formed by the combination of nitrogenous bases A, C, G, and U, sugars, and a phosphate backbone. Loops, kissing loops, bulges, hairpins, and pseudoknots are examples of prominent RNA motifs. In this study, the concentration is on H-type pseudoknots, and specifically those which include hairpins, internal loops, and bulges in their loops.

#### 3.1.1. Pseudoknot Structure

There are different classifications of pseudoknots taking into account their genus and order. Reidys et al. [49] proposed a classification scheme based on the genus and order of pseudoknots:

Genus 1 Pseudoknots (G1):

- These pseudoknots consist of one simple pseudoknot.
- They are characterized by the presence of one crossing arc.
- Examples of G1 pseudoknots include simple hairpin loops with a single stem-loop interaction.

Higher Genus Pseudoknots (HG):

- These pseudoknots have more complex topologies with multiple crossing arcs.
- They are characterized by the presence of more than one crossing arc, indicating higher structural complexity.
- Examples of higher genus pseudoknots include those with multiple stem-loop interactions and interwoven base pairs.

Regarding the order of pseudoknots, Huang et al. [50] and Vernizzi et al. [51] proposed the following classification:

First Order Pseudoknots:

- These are the simplest pseudoknots, typically involving one level of nesting.
- The interactions within these pseudoknots can be described without considering more complex interdependencies.
- First order pseudoknots often include simple stem-loop structures with one intervening loop.

Higher Order Pseudoknots:

- These pseudoknots exhibit more complex nesting patterns and interactions.
- They involve multiple levels of nesting, where the formation of one base pair may depend on the formation of others.
- Higher order pseudoknots can include intricate arrangements of stem-loops and intervening loops, leading to more intricate folding patterns.

**Figure 1:**
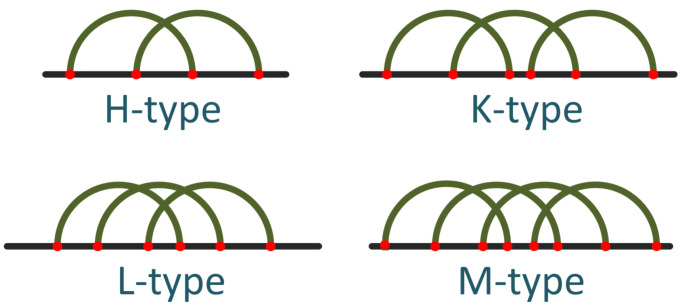
The predominant types of pseudoknots (after [53]).

These classifications offer a framework for understanding the structural diversity of RNA pseudoknots, encompassing both their topological complexity (genus) and nesting patterns (order). Pseudoknots, although infrequent in RNA sequences, present a formidable challenge in terms of prediction. The complex pattern that arises from the intersection of two base pairs was initially noticed in the Turnip Yellow Mosaic virus [52]. The most basic kind of pseudoknot consists of two single-stranded sections; although there are many variants, the most common types are H, K, L, and M, as Figure 1 [53,54] shows. In particular, two stems and two loops of any length characterize the H-type pseudoknot [14]. To create this pseudoknot, two base pairs—which we refer to as core stems in our notation—must formed.

Knotify [20] predicts H-type pseudoknots and its variation, Knotify+ [19], predicts H-type with internal loops and bulges. Knotify for L-type [42], finally, introduces a grammar and a computational pipeline for predicting a rare pseudoknot type, the L-type.

Knotify is a specialized computational tool designed for predicting H-type pseudoknots in RNA structures. Utilizing Stochastic Context-Free Grammar (SCFG), it focuses on maximizing base pairs and minimizing free energy to enhance the accuracy of pseudoknot prediction. Building upon the capabilities of Knotify, the subsequent version, Knotify+, introduces the ability to predict H-type pseudoknots that incorporate internal loops and bulges within their loops. While maintaining the use of SCFG, Knotify+ employs a distinct pipeline that integrates maximum base pairs, minimum free energy, and the Cartesian product approach. Knotify_V2.0 represents a further evolution in this series, partially incorporating the features of its predecessors and introducing additional grammatical rules tailored to hairpin loop prediction (a second grammar). This version offers a sophisticated mechanism for choosing the optimal H-type structure, whether it includes bulges, internal loops, both, or neither.

In the realm of RNA, an analysis of protein secondary structures can be paramount. One notable approach involves utilizing the Secondary Structure Assignment of Protein Fragments (SACF) method. This method facilitates the assignment of protein helices based on their geometric characteristics. The SACF method has been extensively discussed and validated in the literature, with relevant references including [55,56,57]. Additionally, various algorithms have been developed for the assignment of protein helices, leveraging helix geometry as a primary criterion. These algorithms play a crucial role in accurately identifying and characterizing protein secondary structures. For further insights into these methodologies and their applications, pertinent literature should be consulted.

#### 3.1.2. Hairpins in Pseudoknots

**Figure 2:**
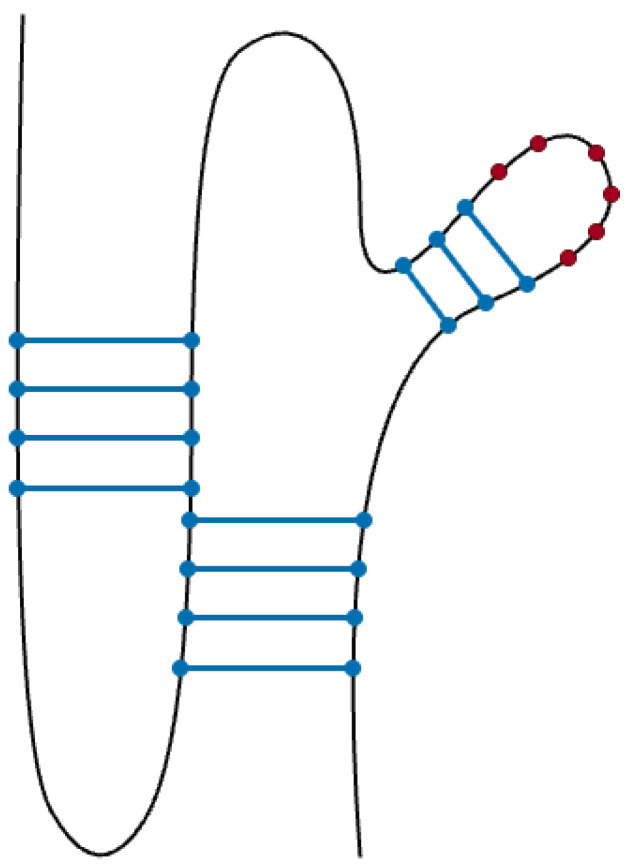
H-type pseudoknot with hairpin in its loop. Hairpin unpaired bases creating a stem-loop structure are represented with red dots, while blue dots represent stems.

Hairpins, crucial structural elements in RNA [58], contribute to the intricate architecture of pseudoknots. These elements are formed when a sequence of unpaired bases on a single-stranded RNA strand folds back and pairs with complementary bases, creating a stem-loop structure. Hairpins play a significant role in stabilizing RNA structures and influencing the overall folding pattern. Similar to bulges and internal loops, the inclusion of hairpins in our pseudoknot prediction framework is motivated by their widespread occurrence in functional RNAs. Figure 2 provides a visual representation of a pseudoknot with incorporated hairpins, highlighting the distinctive stem-loop structures formed by the folding of unpaired bases.

### 3.2. Syntactic Pattern Recognition in Knotify_V2.0 Framework

The proposed computational framework, Knotify_V2.0, represents an advancement of the methodology introduced in [19], now expanded to include the prediction of hairpins in the loops. The fundamental model employed for pseudoknot prediction, specifically of the H-type, relies on the application of context-free grammar (CFG). Within the realm of syntactic pattern recognition theory, a language [59] is initially defined as a collection of syntactic rules. These rules give rise to parse trees that encapsulate the sequence of interest at the terminal nodes. The grammar itself constitutes a set of syntax rules enriched with a designated vocabulary. Following these principles, the framework identifies the adherence of a sequence of symbols to a specific language. As per the framework’s design, Knotify_V2.0 adopts a CFG, a well-established concept categorized by Noam Chomsky [60] within the Chomsky hierarchy. This classification system, dividing grammars into four categories, forms the theoretical underpinning for Knotify_V2.0’s syntactic pattern recognition approach. This approach, rooted in CFG, finds broad application across various domains, including speech processing and compiler construction [61].

Knotify_V2.0, much like the approach presented in [19,20], integrates Yet Another Early Parser (YAEP) [62]. YAEP is a high-performance implementation of Earley’s [63,64,65,66] parser designed for handling ambiguous grammar, making it suitable for our RNA pseudoknot prediction grammar.

## 4. Overview of Our Approach

**Figure 3:**
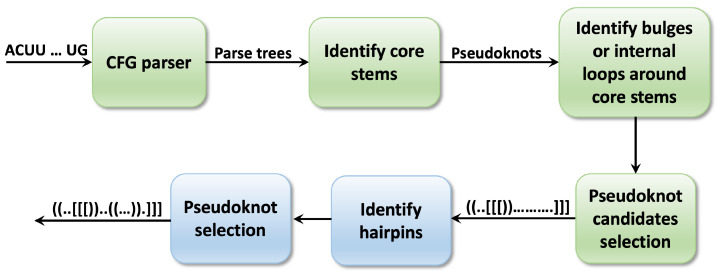
The previous methodology of Knotify+ is highlighted in green, while the extended proposed methodology of Knotify_V2.0 is presented in blue.

This section outlines the methodology introduced by the Knotify_V2.0 platform. It incorporates the pruning technique discussed in [53] and integrates the prediction of bulges and internal loops around the core stems as presented in [19]. The main contribution of the proposed platform is the capability of predicting the existence of hairpins in the pseudoknot loops. The initial version, Knotify, accomplishes pseudoknot prediction within an RNA sequence through a threefold process: (a) using a context-free grammar (CFG) parser to analyze the RNA sequence, generating trees where a pseudoknot motif is identified; (b) analyzing the generated parse trees to identify the two stems that constitute the pseudoknot and potential base pairs proximal to these core stems; (c) selecting the optimal tree based on two established criteria: maximum count of base pairs and minimum free energy of the sequence. An extension of Knotify, Knotify+ [19], augment the prediction capabilities of the platform by identifying bulges and internal loops next to the core stems. Knotify_V2.0, incorporates the module of Knotify+ for bulges and internal loops and introduces two additional tasks (depicted in the blue boxes in Figure 3) following pseudoknot selection, tasked with identifying hairpins and then reapplying the selection criteria. This new procedure proposes an optimized pipeline for a wider range of pseudoknot predictions. The following subsections provide a comprehensive examination of these tasks.

Following previous implementations, the input for the system is a string representing an RNA sequence composed of nitrogenous bases. The output consists of the base pairing information for the provided RNA sequence, presented in an extended dot-bracket notation. Detailed source code and implementation specifics for Knotify_V2.0 are available for reference in a public github repo [67].

### 4.1. Knotify_V2.0 Algorithm

The Knotify algorithm is a sophisticated computational approach designed for the analysis of RNA secondary structures, emphasizing the complexity of H-type pseudoknots and hairpins in their loops. This section delves into the algorithm’s methodology, illustrating its precision in predicting RNA structural motifs.

#### 4.1.1. Example Usage

The Knotify_V2.0 algorithm is executed through a command-line interface, allowing the input of an RNA sequence and the specification of additional parameters and libraries.

#### 4.1.2. Algorithm Stages

The Knotify_V2.0 algorithm (see Algorithm 1) provides a robust and precise tool for anticipating RNA secondary structures. Through its multi-stage process, which involves stem detection, annotation, energy assessment, and hairpin identification, it furnishes molecular biologists and geneticists with a potent resource to comprehend the behavior and structure of RNA. It is modular and can be easily modified to include different criteria and structures depending on the needs, allowing a fast experimentation process.
**Algorithm 1** Knotify_V2.0—The variation of Knotify+ Algorithm for the inclusion of Hairpin loops**Require:** 
RNA sequence *S*, Tuning parameters *P***Ensure:** 
Predicted structure *R*, Free energy *E*, Execution time *D*  1:*# Initialization and Inputs*  2:S← input RNA sequence  3:P← tuning parameters  4:   5:*# Stage 1: Detect Core Stems*  6:Parse *S* using Earley Grammar  7:C← Candidate positions for core stems in *S*  8:   9:*# Stage 2: Annotate Core Stems*10:**for each** candidate *c*** in** *C* **do**11:    Annotate *c* with core stems, considering bulges and/or internal loops12:**end for**13:A← Annotated candidates14: 15:*# Stage 3: Apply Criteria*16:M← Max stems in *A*17:$A^{\prime }\leftarrow$ Filter *A* by stems ≥M18:Compute MFE and sort $A^{\prime }$ in a ascending order19: 20:*# Stage 4: Identify Hairpins*21:**for each** candidate *a* **in** $A^{\prime }$ **do**22:    Identify hairpins in *a*23:    Generate variants *V* with/without hairpins in the loops24:**end for**25: 26:*# Stage 5: Final Selection*27:Reapply max stems and MFE criteria on *V*28: 29:R,E← Candidate with min MFE in *V*, and its energy value30:D← Calculate execution time31: 32:*# Final outputs*33:**return** R,E,D

#### 4.1.3. Stage 1: The Grammar to Locate the Pseudoknots

Knotify_V2.0 is rooted in the framework proposed by [20], leveraging an efficient Context-Free Grammar (CFG) parser. This involves first choosing primitive patterns, where nitrogenous bases G, U, C, and A are represented by the RNA sequences as the characters “G”, “U”, “C”, and “A”, respectively. These characters make up the set T of terminal symbols in the language. These letters indicate an RNA string in sequences like AUGCCAGG or CGGUUACCGA. Primitive patterns were chosen, and a CFG was constructed for syntactic analysis of the language representation of these patterns.

The CFG $G_{RNA}$ that was first introduced and fully explained in [20] was employed, and Knotify_V2.0 implemented a space elimination step proposed in [53] to greatly decrease the number of substrings parsed using a sliding-windows method. After examining the RNA sequence, the CFG parser produced parse trees that identified patterns of pseudoknots. This work’s main contribution is the addition of a new module for hairpin prediction, which is detailed in Section 4.1.4 after the pseudoknot decoration and the prediction of bulges and internal loops proposed in [19]. Finally, the pseudoknot selection task was performed following the methodology described in [53], as outlined in Section 4.1.7.

#### 4.1.4. Stage 2: Core Stems Annotation

In the initial phase, the parser constructed parse trees encompassing all relevant information. Utilizing these trees, the identification of potential pseudoknots and their respective core stems was carried out. Subsequently, the second task involved traversing these parse trees to identify additional stems. The context-free grammar (CFG) was specifically created to identify the main pseudoknot intersecting stems—referred to as core stems in our notation—to improve the CFG parser’s performance. As a result, an analysis of every parse tree sought to identify base pairs encircling the pseudoknot’s central stems. Every base in the two loops was looked at one after the other to see if it might couple with another base that was in the right place.

Table 1 shows how the core stems are decorated. The identification of the two pseudoknot loops came next, after core stems G-C and C-G were found at positions 7–20 and 2–8. 2–6 were covered by the left loop, and 9–19 were covered by the right loop. We carefully examined bases inside these loops to determine whether they could be paired with bases outside of the pseudoknot loops. In particular, base pairs in the right loop were compared for compatibility with the base at position 1, while base pairs in the left loop were evaluated against bases at locations 21 to 22.

During stage 2, sequential detection of base pairs at positions 1–9, 6–21, and 5–22 occurred within both loops of the pseudoknot. A detailed account of this process is provided in Table 1.

#### 4.1.5. Stage 4: Introduce Hairpin Loops in the Pipeline

Upon exhausting the possibility of forming additional sequential base pairs, scrutiny shifted to the presence of bulges and internal loops. Subsequently, an examination of the existence of hairpins was conducted. Unpaired bases on both the left and right pseudoknot loops were scrutinized to assess their potential for forming a base pair, thereby creating a hairpin loop. In the example given, the set at locations 12–13 was considered for possible base pairing with the set at positions 17–18, potentially forming a hairpin with unpaired bases at positions 14–17.

Users may define a tuning parameter to include candidates with fewer stems, e.g., if the maximum number of stems is 9, we opt to include candidates with 7 stems or more. Candidates are then sorted based on the minimum free energy (MFE) of their structure. For each candidate, another grammar $G_{hairpins}$ in the right and left loops is employed to identify matching pairs conducive to hairpin formation. If a hairpin is detected, four variations of each candidate are considered: without a hairpin, with a hairpin in the right loop (if any), with a hairpin in the left loop (if any), and with a hairpin in both loops (if any). Finally, the max stems/min free energy criterion is reapplied, now incorporating the presence of hairpin stems. The algorithm’s final output is the structure with the minimum calculated MFE. Grammar $G_{hairpins}$ is described in next section.

#### 4.1.6. Description of the Grammar $G_{hairpins}$ for RNA Hairpin Motifs

The presented grammar (see Table 2) is designed to model the formation of hairpin motifs in RNA sequences, essential structures influencing RNA function and interactions. These motifs consist of a stem, formed by complementary base pairs, and a loop of unpaired nucleotides. The grammar employs a formal system to generate such structures, detailed as follows.

- **Terminals:** The nucleotides adenine (A), uracil (U), cytosine (C), and guanine (G) are represented by ‘a’, ‘u’, ‘c’, ‘g’, respectively.
- **Non-terminals:** Symbols such as S, P, L, R, M, K represent different structural components. L and R denote the stem’s left and right parts, while P represent the loop or a sequence contributing to both stem and loop.
- **Syntax Rules:**
  1. S → L P R encapsulates the overall structure of a hairpin motif, with S representing the entire structure composed of a left stem part (L), a loop or connecting region (P), and a right stem part (R).
  2. The P rules define the loop formation or how nucleotides can pair up in the stem part.
  3. The L, R, M → K rules suggest that both stem sides and some other component (M) are formed by sequences described by K, implying a recursive structure for the stem.
  4. K rules allow for any combination of the four bases and potentially empty sequences (ϵ), crucial for modeling the variable length of RNA stems and loops.

This grammar captures the essential features of hairpin formation in RNA, including the variable length and composition of both the stem and loop regions, accommodating the complexity of RNA structural biology.

#### 4.1.7. Stage 3, 5: Optimal Tree Selection

Knotify_V2.0 adopted a comprehensive strategy to choose the optimal tree among those produced by the CFG, intending to improve base pairing while adhering to the Minimum Free Energy (MFE) principle. Initially, the system organized the trees based on the count of base pairs surrounding the core stems of the pseudoknot, taking into account stems that formed after bulges or internal loops. Subsequently, in the succeeding stage, the system applied the MFE criteria to the top-ranked trees from the initial phase, particularly focusing on those exhibiting the highest number of base pairs in proximity to the pseudoknot. Then, the system identifies hairpin loops in the loops of the pseudoknots, creating a set of new variations of structures, with some of them containing hairpins in the loops and others not. In that set of candidates, the maximum stem criterion is applied, and the ones with the most base pairs proceed to the final stage. Ultimately, the structure with the minimum free energy will be selected from the available options. A module, adapted from HotKnots [68], evaluated each candidate’s energy, thereby supplying energy scores to Knotify_V2.0 for the final decision-making. This energy evaluation method was originally proposed by Mathews [69], but in this work, a variation from [70] was used.

## 5. Evaluating Performance

### 5.1. Construction of Datasets

To evaluate the precision of Knotify_V2.0 in comparison to alternative methodologies, a dataset [71] consisting of 20 renowned RNA sequences, each containing pseudoknots, was compiled. In all these sequences, hairpins were present on either one or both sides of the pseudoknot’s core stems. The RNA sequences were sourced from public databases, the RNA Database platforms [72,73]. The comparative analysis involved our proposed methodology and five efficient implementations from the literature, namely IPknot, Knotty, Hotknots, IHfold, and IHfoldv2 [29,32], together with the previous version of our implementation. This resulted in the inclusion of seven platforms in the performance evaluation, namely IPknot, Knotty, Hotknots, IHfold, IHfoldv2, Knotify+, and Knotify_V2.0.

### 5.2. Evaluation Methods

Our framework’s performance was evaluated through three methods: (a) determining the percentage of accurately predicted pseudoknot core stems; (b) examining the confusion matrix, which included precision (PPV), recall, F1-score, and MCC (Matthews correlation coefficient); and (c) assessing the execution time.

#### 5.2.1. Prediction of Pseudoknots’ Core Stems

Table 3 provides a summary of each platform’s ability to predict pseudoknot core stems. The second column indicates the number of pseudoknots for which a platform accurately predicted both core stems, while the fourth column displays the number of pseudoknots for which a platform correctly predicted only one core stem. Similar to Knotify+, Knotify_V2.0 successfully identified both pseudoknot core stems in 14 out of 20 sequences. On the contrary, IPknot achieved this in 2 sequences, Hotknots in 6 sequences, and Knotty and IHfoldv2 in 4 sequences each.

While some platforms managed to additionally predict one core stem of the pseudoknot in certain sequences, Knotify_V2.0 exhibited exceptional performance by correctly identifying a minimum of one core stem in 70% of the sequences in the dataset. On the contrary, IPknot achieved this in 25%, Knotty in 35%, Hotknots in 35%, IHfold in 0%, IHfoldv2 in 35%, and Knotify+ in 60% of the sequences. This result highlights that Knotify_V2.0 outperformed other popular platforms, including Knotify+, our earlier implementation, and even in situations where obtaining a precise prediction was difficult.

We adopted the pseudoknot finding methods described in [69] and permitted the displacement of one base per stem, either left or right, by one position. As a result, it was decided that the combination (*k*, *l*) was equal to either (*k* ± 1, *l*) or (*k*, *l* ± 1).

**Figure 4:**
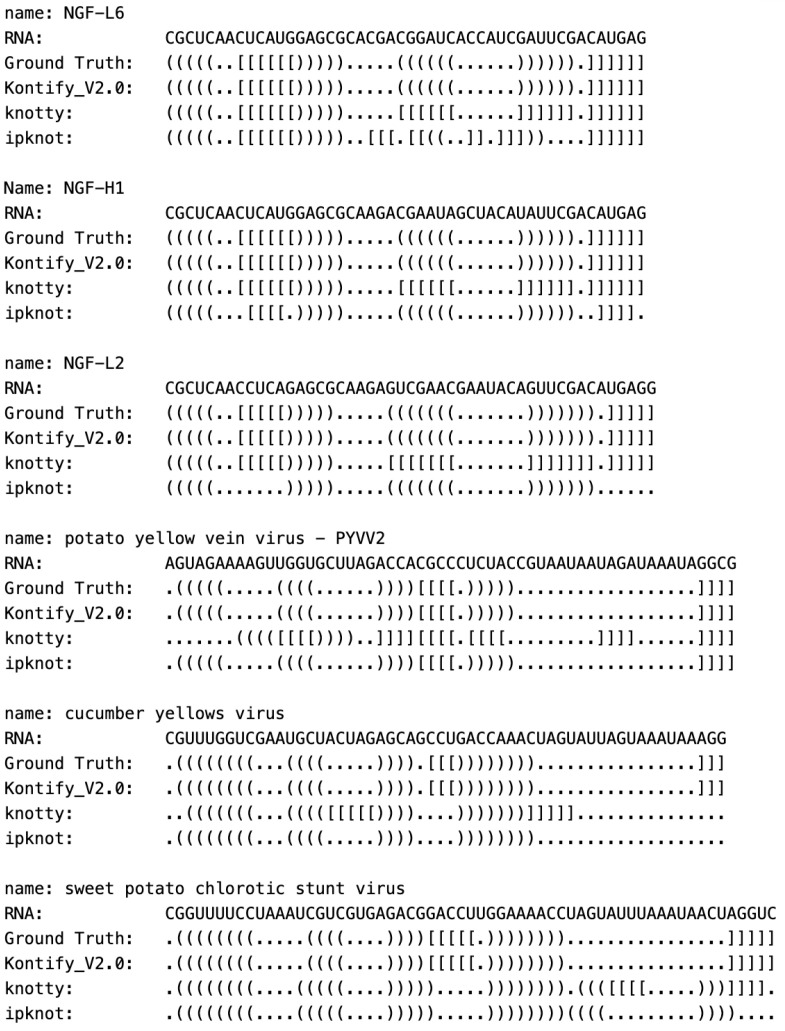
Representative case studies.

Figure 4 illustrates case studies that showcase instances where Knotify_V2.0 demonstrates superior performance compared to the other two models, which exhibited performance levels similar to the proposed model. Evidently, the proposed methodology effectively identifies the core stems of the pseudoknot and a hairpin structure when situated on the right loop of the pseudoknot (cases: NGF-L6, NGF-H1, NGF-L2). Furthermore, the proposed methodology efficiently detects the core stems of the pseudoknot and a hairpin structure when located on the left loop of the pseudoknot (cases: potato yellow vein virus—PYVV2, cucumber yellows virus, sweet potato chlorotic stunt virus).

**Figure 5:**
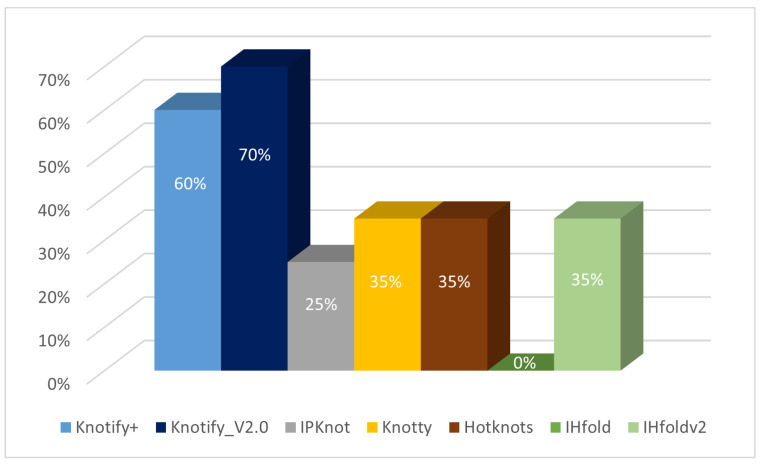
The percentage of each method’s core stem predictions.

The results of estimating the core stems of the pseudoknots are shown in Figure 5.

#### 5.2.2. Confusion Matrix, Precision, Recall, F1-Score, and MCC

A summary of each platform’s performance in terms of precision, recall, F1-score, and the Matthews Correlation Coefficient (MCC) can be found in Table 4. The Formulas (1)–(4) provide an explanation of each of these measures. The counts of correctly predicted base pairs are represented by *tp* (true positive), incorrectly predicted base pairs by fp (false positive), base pairs that were not predicted by *fn* (false negative), and base pairs that were correctly not predicted by *tn* (true negative) in these equations.

(1) $$PPV=\frac{tp}{tp+fp}$$

(2) $$Recall=\frac{tp}{tp+fn}$$

(3) $$F1-score=\frac{2\times PPV\times Recall}{PPV+Recall}$$

(4) $$MCC=\frac{tp\times tn-fp\times fn}{\sqrt{\left(tp+fp)(tp+fn)(tn+fp)(tn+fn\right)}}$$

The analysis of the algorithms presented in the confusion matrix for the complete dataset reveals distinct performances across various metrics without directly focusing on the counts of true positives, false positives, false negatives, and true negatives. As indicated in Table 4, the suggested implementation demonstrated superior performance compared to the previous version, Knotify+, across all metrics. Knotify_V2.0 achieved a higher count of true positives (*tp*), and a significantly lower count of false negatives (*fn*) compared to Knotify+, underscoring improvements in prediction and recall, respectively. Knotify_V2.0 emerges as a notably efficient system in identifying relevant instances, as indicated by its leading F1-score, which in turn justify its overall prediction rate and effectiveness. This efficiency is a result of the algorithm’s exceptional ability to recall, balanced by its reasonable precision. On the other hand, Knotty and Hotknots also show commendable performances, but with slight variations in precision and recall, which affects their respective F1-scores and overall efficiency in identifying positive instances.

Comparatively, Knotify_V2.0’s recall rate is approximately 11% and 10% higher than Knotty’s and Hotknots’, respectively, highlighting its superior capability to identify positive instances. Despite this, Knotty holds a slight edge over Knotify_V2.0 in precision by about 2%, reflecting a marginally better accuracy in classifying positive instances correctly. When considering the F1-score, which captures the balance between precision and recall, Knotify_V2.0 surpasses Knotty and Hotknots by 4% and 6%, respectively. This indicates that despite facing challenges with precision, Knotify_V2.0 maintains a balanced and effective performance across the metrics. Considering the IPknot algorithm, it is quite competitive, but it falls slightly behind these state-of-the-art methodologies in each assessed metric.

#### 5.2.3. Execution-Time Comparison

The execution time was taken into consideration as the third parameter, which was used to compare the suggested methodology’s time efficiency to that of other platforms. Each method’s prediction time is shown in Table 5.

In the second column of Table 5, the per-platform execution time for the entire dataset is presented. Specifically, Knotify_V2.0 required 37.93 s, IPknot required 8.78 s, Knotty required 26.37 s, Hotknots required 6.01 s, IHfold required 0.68 s, and IHfoldv2 required 1.75 s. Notably, Knotify_V2.0 exhibited a slightly longer execution time compared to Knotty, because of the introduction of the new module for the hairpin motif. The third column displays the average execution time for each method.

## 6. Conclusions

Anticipating the secondary structures of RNA, especially pseudoknotted ones, presents a substantial challenge. To address this, the proposed framework introduced an intelligent grammar-based algorithm specifically designed for predicting H-type pseudoknots, including hairpins in their loops. This algorithm displayed effective detection of secondary structures, demonstrating accuracy comparable to and in many metrics higher than those of established platforms. Knotify_V2.0 performs better than its predecessor Knotify+, surpassing it in recall and F1-score. Knotify_V2.0 exhibited superior accuracy in predicting core stems compared to state-of-the-art frameworks. It delivered exceptional performance, accurately identifying the core stems of the pseudoknot in 70% of the sequences. Consequently, Knotify_V2.0 attained a higher F1-score than any other platform. Despite being approximately 0.7 times (26.37/37.93 = 0.69) slower than Knotty, Knotify_V2.0 consistently maintained the highest percentage in accurately predicting core stems across all scrutinized methods. In future work, modifications to enhance Knotify_V2.0, enabling the prediction of more complex RNA secondary structures, including K and M-type pseudoknots and combined motifs, will be implemented. In this direction, the research team will refine the algorithmic approach and grammar to handle increased structural complexity, providing a more comprehensive tool for RNA research. Additionally, a web-based platform will be developed, which will encapsulate various system versions. This platform will allow for easy selection of appropriate parameters and interaction through a user-friendly interface, facilitating broader access and utility. Furthermore, the development of a hybrid system that combines the robust features of Knotify_V2.0 with advanced machine-learning techniques will be evaluated. This initiative aims to leverage the strengths of grammar-based and AI approaches, potentially leading to significant improvements in prediction accuracy and computational efficiency. By advancing these areas, substantial contributions to the research of RNA structure prediction are anticipated, supporting more detailed and accurate biological insights.

## Figures and Tables

**Table 1:** The process of decorating an H-type pseudoknot’s core stems. Red parenthesis indicates paired bases that create a hairpin.

**Base Index**	1	2	3	4	5	6	7	8	9	10	11	12	13	14	15	16	17	18	19	20	21	22
RNA	U	C	A	A	A	U	G	G	A	C	A	U	A	G	C	A	U	A	A	C	A	U
Stage 1:	.	(	.	.	.	.	[	)	.	.	.	.	.	.	.	.	.	.	.	]	.	.
Stage 2	.	(	.	.	.	[	[	)	.	.	.	.	.	.	.	.	.	.	.	]	]	.
Stage 2	.	(	.	.	[	[	[	)	.	.	.	.	.	.	.	.	.	.	.	]	]	]
Stage 2	(	(	.	.	[	[	[	)	)	.	.	.	.	.	.	.	.	.	.	]	]	]
Stage 4	(	(	.	.	[	[	[	)	)	.	.	(	(	.	.	.	)	)	.	]	]	]

**Table 2:** Description of the Grammar $G_{hairpins}$.

#	Syntactic Rules
1	S → L P R
2	P → ‘a’ P ‘u’
3	P → ‘u’ P ‘a’
4	P → ‘g’ P ‘c’
5	P → ‘c’ P ‘g’
6	P → ‘g’ P ‘u’
7	P → ‘u’ P ‘g’
8	P → M
9	L → K
10	R → K
11	M → K
12	K → ‘a’ K
13	K → ‘u’ K
14	K → ‘c’ K
15	K → ‘g’ K
16	K →ϵ

**Table 3:** Prediction of pseudoknot position based on core stems throughout the entire dataset.

Platform	2 Matches	2 Matches (%)	1 Match	At Least 1 Match (%)
IPknot	2	10	3	25
Knotty	4	20	3	35
Hotknots	6	30	1	35
IHfold	0	00	0	0
IHfoldv2	4	20	3	35
Knotify+	11	55	1	60
Knotify_V2.0	14	70	0	70

**Table 4:** Each Algorithm’s confusion matrix for the complete dataset.

Algorithm	tp	tn	fp	fn	Precision	Recall	F1-Score	MCC
IPknot	540	102	425	152	0.560	0.780	0.652	−0.032
Knotty	518	212	366	123	0.586	0.808	0.679	0.196
Hotknots	534	144	418	123	0.561	0.813	0.664	0.083
IHfold	484	118	431	186	0.529	0.722	0.611	−0.072
IHfoldv2	364	110	430	315	0.458	0.536	0.494	−0.271
Knotify+	292	150	439	338	0.399	0.463	0.429	−0.287
Knotify_V2.0	628	62	461	68	0.577	0.902	0.704	0.033

**Table 5:** The time taken for execution by each algorithm across the entire dataset.

Algorithm	Total Time (s)	Average Time (s)
IPknot	8.78	0.44
Knotty	26.37	1.32
Hotknots	6.01	0.30
IHfold	0.68	0.02
IHfoldv2	1.75	0.09
Knotify+	2.50	0.12
Knotify_V2.0	37.93	1.89

## Data Availability

Dataset available on request from the authors.

## Abbreviations

The following abbreviations are used in this manuscript:

AI	Artificial Intelligence
CFG	Context-free grammar
CCJ	Chen–Condon–Jabbari
CNN	Convolutional Neural Network
CYK	Cocke–Younger–Kasami
DNA	Deoxyribonucleic acid
IBPMP	Improved base-pair maximization principle
LSTM	Long short-term memory
MCC	Matthews correlation coefficient
MFE	Minimum free energy
NP	Nondeterministic polynomial
RNA	Ribonucleic acid
SCFG	Stochastic context-free grammar
YAEP	Yet another early parser
